# Identification of Chebulinic Acid and Chebulagic Acid as Novel Influenza Viral Neuraminidase Inhibitors

**DOI:** 10.3389/fmicb.2020.00182

**Published:** 2020-02-28

**Authors:** Ping Li, Ruikun Du, Yanyan Wang, Xuewen Hou, Lin Wang, Xiujuan Zhao, Peng Zhan, Xinyong Liu, Lijun Rong, Qinghua Cui

**Affiliations:** ^1^College of Pharmacy, Shandong University of Traditional Chinese Medicine, Jinan, China; ^2^Qingdao Academy of Chinese Medicinal Sciences, Shandong University of Traditional Chinese Medicine, Qingdao, China; ^3^Research Center, College of Chinese Medicine, Shandong University of Traditional Chinese Medicine, Jinan, China; ^4^Department of Medicinal Chemistry, Key Laboratory of Chemical Biology (Ministry of Education), School of Pharmaceutical Sciences, Shandong University, Jinan, China; ^5^Department of Microbiology and Immunology, College of Medicine, University of Illinois at Chicago, Chicago, IL, United States

**Keywords:** influenza A virus, chebulinic acid, chebulagic acid, neuraminidase, neuraminidase inhibitor

## Abstract

The influenza A virus (IAV) causes seasonal epidemics and occasional but devastating pandemics, which are of a major public health concern. Although several antiviral drugs are currently available, there is an urgent need to develop novel antiviral therapies with different mechanisms of action due to emergence of drug resistance. In this study, two related compounds, chebulagic acid (CHLA) and chebulinic acid (CHLI), were identified as novel inhibitors against IAV replication. A reporter virus-based infection assay demonstrated that CHLA and CHLI exhibit no inhibitory effect on IAV entry or RNA replication during the virus replication cycle. Results of viral release inhibition assay and neuraminidase (NA) inhibition assay indicated that CHLA and CHLI exert their inhibitory effect on the NA-mediated viral release. Moreover, oseltamivir-resistance mutation NA/H274Y of NA is susceptible to CHLA or CHLI, suggesting a different mechanism of action for CHLA and CHLI. In summary, CHLA and CHLI are promising new NA inhibitors that may be further developed as novel antivirals against IAVs.

## Introduction

Influenza vaccines and antiviral drugs are effective in preventing infection or ameliorating disease severity (Vasileiou et al., 2017). However, influenza infection, as an acute respiratory disease caused by seasonal outbreaks and, periodically, pandemics of influenza viruses and accounting for up to 650,000 annual deaths globally (World Health Organization [WHO], 2018), remains a serious public health concern. This is partly due to the fact that current influenza vaccines can target only selected strains based on annual surveillance and prediction, which does not always match the circulating strains, leading to a sharp drop in vaccine efficacy (de Jong et al., 2000; Nachbagauer and Krammer, 2017). Moreover, emergence of drug resistance strains of influenza A viruses (IAVs) reduces the effectiveness of the current anti-influenza therapies. The three clinically available classes of antivirals for treatment and prevention of influenza infections are the viral ion channel M2 blockers (amantadine and rimantadine), neuraminidase (NA) inhibitors (oseltamivir, zanamivir, peramivir, and laninamivir), and most recently the licensed baloxavir marboxil, which is an oral cap-dependent endonuclease inhibitor of influenza virus polymerase inhibitor (Alves Galvao et al., 2014; Alame et al., 2016; Kashiwagi et al., 2016; Heo, 2018; Noshi et al., 2018). However, high levels of resistance to M2 channel blockers and NA inhibitors have emerged, undermining the efficacy of these drugs (Hata et al., 2007; Samson et al., 2013; van der Vries et al., 2013; Lackenby et al., 2018). Moreover, mutations responsible for reduced susceptibility of influenza A/H3N2 virus to baloxavir have been detected already (Takashita et al., 2019). Therefore, the development of antiviral drugs with different mechanisms of action is urgently needed against influenza viruses.

Natural products contain structurally diversified bioactive chemicals, which are valuable sources for new drug discovery. Among the new medicines approved by the U.S. Food and Drug Administration (FDA) between 1981 and 2010, natural products or their direct derivatives account for 34% (Harvey et al., 2015). In the present study, 352 natural product samples were screened for anti-IAV activity using a phenotypic screening approach based on a recombinant IAV expressing Gaussia luciferase (Zhao et al., 2019). The extracts from both unripe and ripe pods of *Terminalia chebula* Retz. display potent anti-IAV activity. Further, two constitutes of *T. chebula*, chebulagic acid (CHLA) and chebulinic acid (CHLI), were identified as novel antivirals against IAV. Detailed studies revealed that both compounds specifically block progeny virus release by inhibiting IAV NA activity. Moreover, CHLA and CHLI showed highly inhibitory efficacy against an oseltamivir-resistant IAV strain, suggesting their potential as novel cost-effective NA inhibitors for controlling influenza virus infections.

## Materials and Methods

### Library of Natural Product Samples

Dispensing granules of 352 natural product samples were purchased from EFONG Pharmaceutical Company (Foshan, Guangdong, China) and arrayed in 96-well plates at a 20-mg/mL stock concentration in DMSO. All of the sample plates were stored at −80°C until use.

### Cells, Viruses, and Compounds

Madin–Darby canine kidney (MDCK) epithelial cells were grown in Dulbecco’s modified Eagle’s medium (DMEM; Cellgro, Manassas, VA, United States) supplemented with 10% fetal bovine serum (FBS; Gibco, Carlsbad, CA, United States), 1000 units/mL of penicillin, and 100 μg/mL of streptomycin (Invitrogen, Carlsbad, CA, United States). Infections were performed in Opti-MEM containing 2 μg/mL *N*-tosyl-L-phenylalanine chloromethyl ketone (TPCK)–trypsin (Sigma–Aldrich, St. Louis, MO, United States). All cells were grown at 37°C in 5% CO_2_.

Recombinant reporter virus PR8-PB2-Gluc and influenza strains A/Puerto Rico/8/1934 (H1N1, A/H1N1/PR8) and A/Wyoming/3/03 (H3N2, A/H3N2/NY) were prepared and stocked in our lab as previously described (Zhao et al., 2018; Wang et al., 2019). Oseltamivir-resistant influenza A/H1N1/pdm(09) virus containing NA/H274Y was provided by Beijing CDC, China, influenza A/Brisbane/10/2007(H3N2) was provided by Chinese Academy of Medical Sciences, and influenza B-Yamagata-like and B-Victoria-like strains were provided by Shandong CDC (Jinan, China).

Fifteen chemical components (> 98% purity) from *T. chebula* were purchased from the National Institutes for Food and Drug Control (Beijing, China).

### High-Throughput Screen

The library of natural product samples was screened using a phenotypic screening approach described previously (Zhao et al., 2019). Briefly, MDCK cells were seeded in 96-well plates at a density of 5000 cells/well 24 h before infection. In the presence of natural product samples (final concentration of 25 μg/mL), MDCK cells were challenged with recombinant reporter virus PR8-PB2-Gluc at an MOI of 0.01. Infection was quantified after 36 h of incubation by measuring the luciferase activity with PierceTM Gaussia Luciferase Glow Assay kit (Thermo Fisher, Hillsboro, OR, United States) according to the manufacturer’s instructions. Data were normalized to signals from the negative controls (virus alone with DMSO), and an average of > 90% inhibition for duplicates was applied for picking hits.

The selected active samples were then reformatted into new 96-well plates and tested against PR8-PB2-Gluc at 25 μg/mL in 0.125% DMSO (v/v) to confirm the primary results. Cell cytotoxicity was examined 48 h post-treatment using the CellTiter-Glo^®^ Luminescent Cell Viability Assay (Promega, Madison, WI, United States), treated for the antiviral screen.

The confirmed hit samples were twofold serially diluted, respectively, for dose–response analysis, and the IC_50_ and CC_50_ values were determined by fitting dose–response curves with a four-parameter logistic regression to the data in GraphPad Prism software (version 5.02, La Jolla, CA, United States).

### One-Cycle Infection Inhibition Assay

To determine whether the hit natural product samples or its derivatives were target to viral entry or genome replication steps, a one-cycle infection inhibition assay was carried out using the reporter virus PR8-PB2-Gluc. Briefly, MDCK cells growing in a 96-well plate were infected with PR8-PB2-Gluc at an MOI of 0.1 in the presence of various concentrations of test samples/compounds. After 1 h of incubation, unabsorbed viruses were removed, and the cells were treated with the tested samples. In order to prevent the second round of infection, DMEM containing 10% FBS, instead of Opti-MEM containing 2 μg/mL of TPCK–trypsin, was used during infection to avoid HA cleavage and infectious virus production. After 24 h, infections were quantified by measuring the luciferase activity with PierceTM Gaussia Luciferase Glow Assay kit (Thermo Fisher, Hillsboro, OR, United States). Meanwhile, in the presence of the reporter virus PR8-PB2-Gluc, cell cytotoxicity for the test compounds was determined.

### Virus Release Inhibition Assay

To determine whether the hit natural product samples or the derivatives target to the release of progeny viruses, MDCK cells were infected with the reporter virus PR8-PB2-Gluc at an MOI of 0.1. At 20 h post-infection (p.i.), the culture medium was removed, and the cells were washed with PBS three times. Fresh Opti-MEM containing 2 μg/mL of TPCK–trypsin was added and cultured for 2 h in the presence of various concentrations of test samples/compounds. The culture medium was harvested and titrated using luciferase assay.

### Neuraminidase (NA) Inhibition Assay

Neuraminidase inhibition assay was performed using Neuraminidase Inhibitors Screening kit (Beyotime Biotechnology, Shanghai, China) according to manufacturer’s instructions. Briefly, NA and various concentrations of test samples/compounds were added to each well of 96-well plates. In order to fully interact between the compounds and NA, the 96-well plates were mixed for 1 min and incubated at 37°C for 2 min. Then fluorescent substrates were added, mixed, and incubated. After incubation for 1 h, the decrease in fluorescence was monitored to reflect NA inhibition efficacy.

### Viral Yield Reduction Assay

Viral yield reduction assay was performed as previously described (Zhu et al., 2011). Briefly, MDCK cells growing in 24-well plates were infected with A/H1N1/PR8, A/H3N2/NY, oseltamivir-resistant influenza A/H1N1/pdm(09), A/H3N2/Brisbane, B-Yamagata-like, and B-Victoria-like strains at an MOI of 0.01 with or without various concentrations of test samples/compounds. The culture supernatants were harvested at 24 h p.i., and virus titers (TCID_50_/mL) in the culture supernatants were determined using MDCK cells.

## Results

### Extracts of *T. chebula* Inhibit IAV Replication

To discover novel antiviral actives against IAV, a library consisting of 352 natural product samples was prepared and screened using a phenotypic screening approach based on a reporter influenza A PR8-PB2-Gluc virus (Zhao et al., 2019) in a primary screen and confirmation screen. As a result, 12 hit samples were identified to inhibit IAV infection (Supplementary Table S1).

**FIGURE 1:**
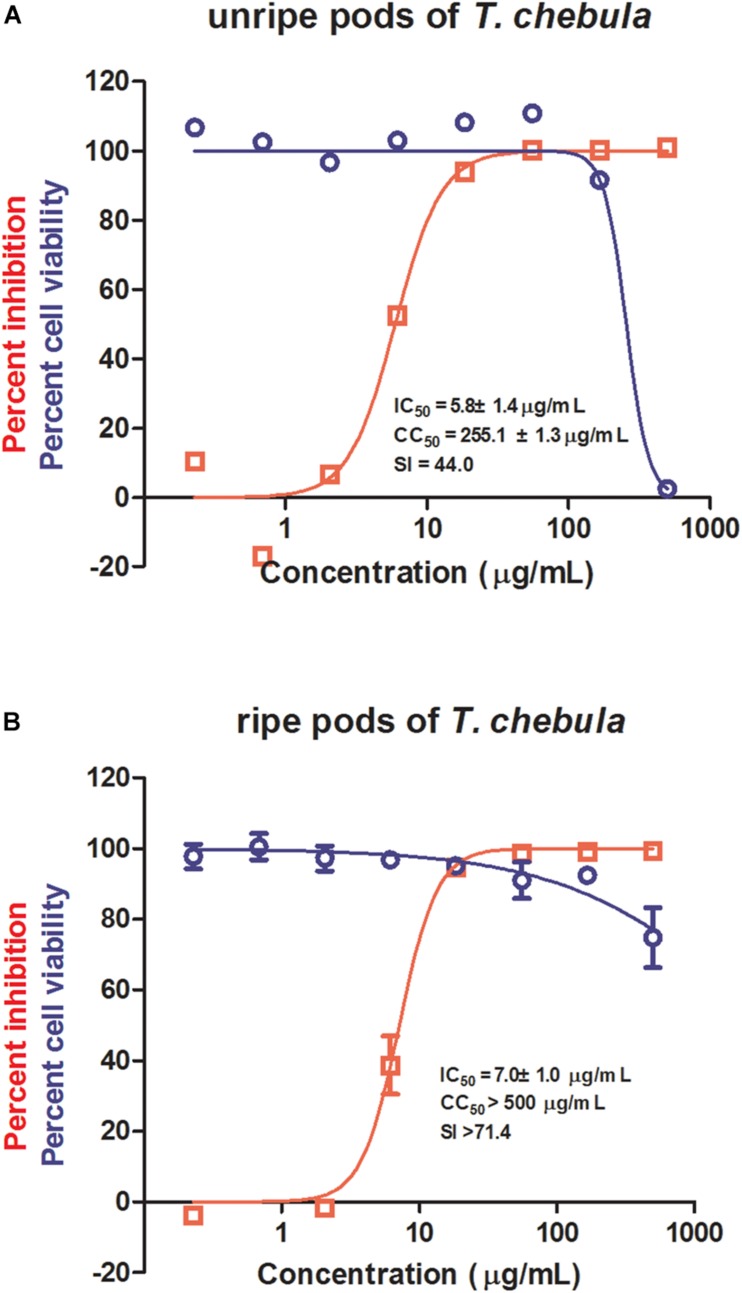
Antiviral activities of unripe and ripe pods of *Terminalia chebula* on influenza virus. The *in vitro* dose–response curves against influenza A virus (IAV) (red) and cytotoxicity in Madin–Darby canine kidney (MDCK) cells (blue) of unripe **(A)** and ripe **(B)** pods of *T. chebula*. Data are means ± SD from three independent experiments.

Among the most potent anti-IAV natural product samples, both unripe and ripe pods of *T. chebula* significantly inhibited IAV replication with IC_50_s of 5.8 ± 1.4 and 7.0 ± 1.0 μg/mL, respectively (Figures 1A,B). Since unripe pods of *T. chebula* exhibited a CC_50_ to MDCK cells of 255.1 ± 1.3 μg/mL, while ripe pods of *T. chebula* showed no obvious cytotoxicity at a high concentration as 500 μg/mL, it is likely that there is some difference between the compositions of the two natural product sample preparations.

### CHLA and CHLI Inhibit IAV Replication

**FIGURE 2:**
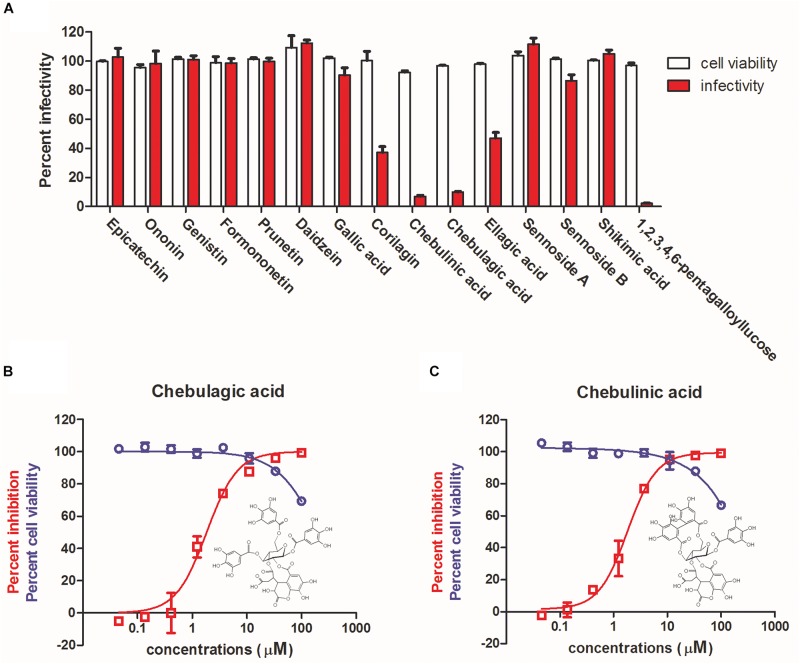
Identification of chebulagic acid (CHLA) and chebulinic acid (CHLI) as novel antiviral actives against influenza virus infection. **(A)** Antiviral determination of 15 overlapped components of unripe and ripe pods of *T. chebula* at 12.5 μM. **(B)** CHLA and **(C)** CHLI were examined against influenza virus in MDCK cells *in vitro* for dose–response evaluation (red) and cytotoxicity assay (blue). Insets: molecular structures of **(B)** CHLA and **(C)** CHLI. Data are means ± SD from three independent experiments.

Considering the close relation between unripe and ripe pods of *T. chebula*, the chemical compositions of the two samples were compared based on spectroscopic data with previously reported literature. Fifteen overlapped constituents were subsequently shortlisted and subjected to antiviral evaluation. As shown in Figure 2A, at a concentration of 12.5 μM, corilagin and ellagic acid showed about 40% inhibition, while CHLA, CHLI, and 1,2,3,4,6-pentagalloyllucose (PGG) showed > 90% inhibition. Since corilagin, ellagic acid, and PGG have been well characterized for their antiviral activity against IAV (Liu et al., 2011; Chen et al., 2015; Zu et al., 2015), CHLA and CHLI, which are structurally related, were chosen for further analysis.

High-performance liquid chromatography was performed to further demonstrate that unripe and ripe pods of *T. chebula* share the constituents of CHLA and CHLI (Supplementary Figure S1), of which the dose–response effects against IAV replication were subsequently determined. As shown in Figures 2B,C, the IC_50_ values against IAV replication of CHLA and CHLI were 1.36 ± 0.36 and 1.86 ± 0.98 μM, respectively, while the CC_50_ values of both compounds were greater than 100 μM. These results show that CHLA and CHLI are potent anti-IAV inhibitors.

### CHLA and CHLI Do Not Inhibit Virus Entry or RNA Replication of IAV

To identify the target(s) and possible mechanism of action for CHLA and CHLI on IAV replication, a reporter IAV-based one-cycle infection assay was performed. Briefly, MDCK cells were infected by the reporter IAV PR8-PB2-Gluc in the absence or presence of test compounds, without the addition of TPCK–trypsin in the culture medium. Without exogenous trypsin-mediated HA cleavage, the progeny viruses are non-infectious and could not initiate a second round of infection. In this context, decreased expression of the reporter luciferase would be expected upon treatment with inhibitors targeting virus entry or RNA replication.

**FIGURE 3:**
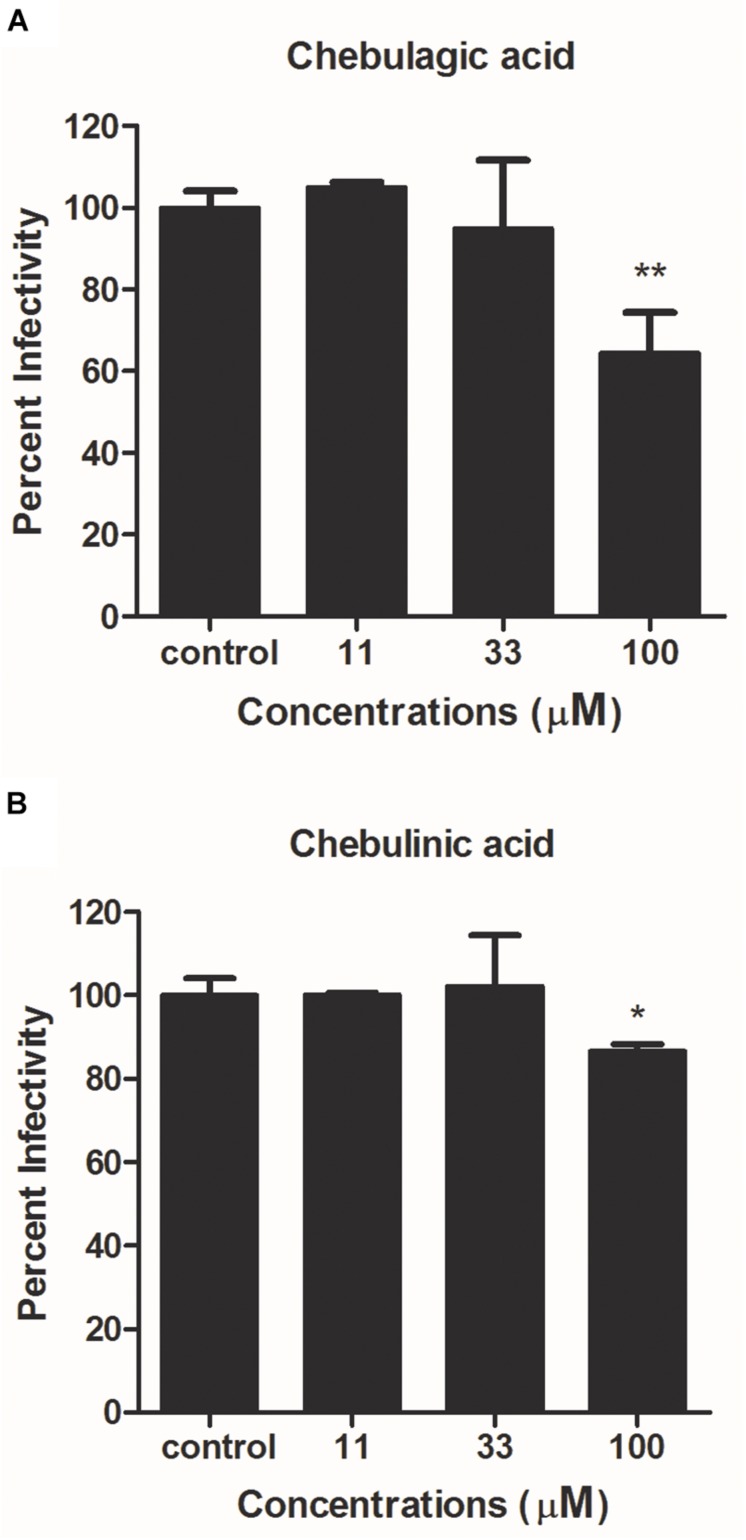
Reporter luciferase-based one-cycle infection inhibition assay. MDCK cells growing in 96-well plates were infected with reporter influenza PR8-PB2-Gluc virus at an MOI of 0.1, in the absence or presence of increasing concentrations of **(A)** CHLA and **(B)** CHLI. Dulbecco’s modified Eagle’s medium (DMEM) supplemented with 4% fetal bovine serum (FBS) were used as culture medium during infection to avoid generation of infectious progeny viruses. At 24 h post infection (p.i.), Gluc expression was determined, and viral infectivities were evaluated. Data are means ± SD from three independent experiments. *p < 0.05; **p < 0.01; Student’s *t*-test.

As shown in Figure 3, neither CHLA nor CHLI reduced Gluc expression. Note that the slight decrease in Gluc levels at 100 μM of CHLA and CHLI might be due to cytotoxicity (Supplementary Figure S2). These results suggest that CHLA and CHLI do not interfere with IAV entry or RNA replication.

### CHLA and CHLI Target NA to Block Viral Release

**FIGURE 4:**
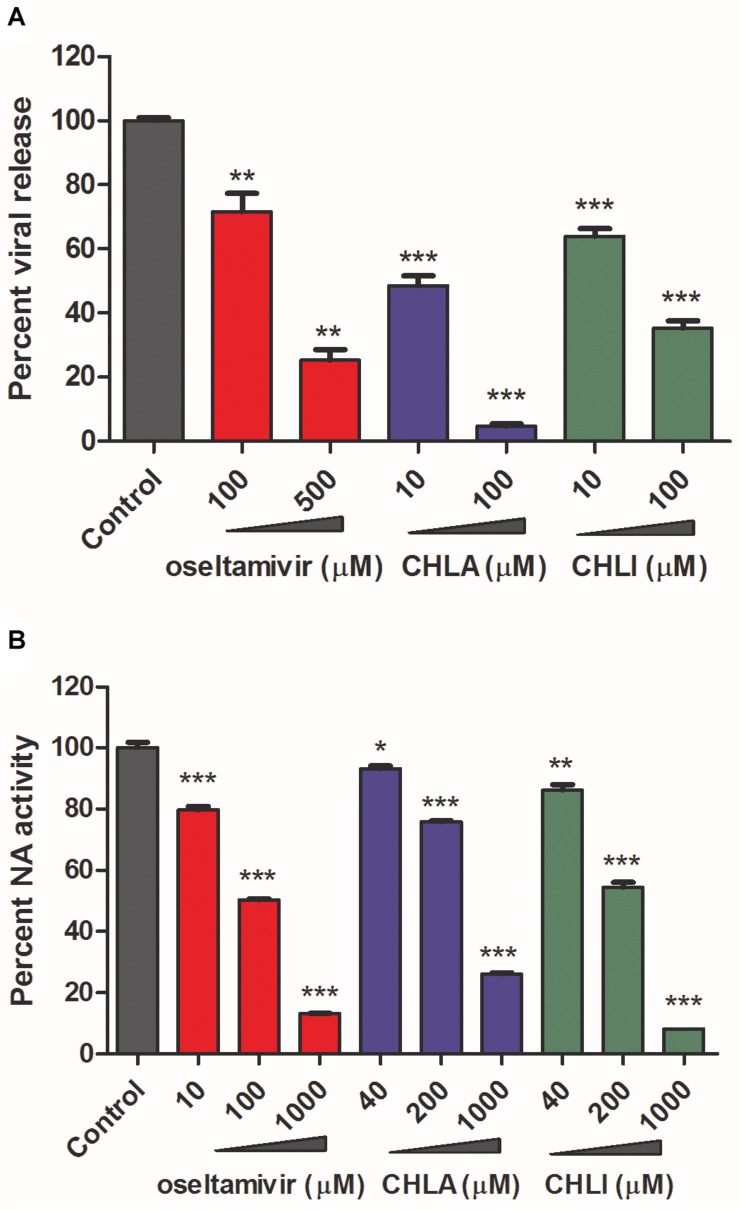
CHLA and CHLI block neuraminidase (NA)-mediated virus release. **(A)** Virus release inhibition assay. MDCK cells were infected with reporter influenza PR8-PB2-Gluc virus at an MOI of 0.1. At 20 h p.i., infected cells were washed three times, and fresh Opti-MEM containing 2 μg/mL of *N*-tosyl-L-phenylalanine chloromethyl ketone (TPCK)–trypsin was added for another 2-h incubation, in the absence or presence of indicated compounds. The supernatants were harvested, and progeny viruses were titrated by luciferase assay. Data are means ± SD from three independent experiments. **p < 0.01; ***p < 0.001; Student’s *t*-test. **(B)** NA inhibition assay. The NA was incubated with fluorimetric substrates in the absence or presence of indicated compounds. The intensity of fluorescence was detected to reflect NA activities. Data are means ± SD from three independent experiments. *p < 0.05; **p < 0.01; ***p < 0.001; Student’s *t*-test.

The effects of CHLA and CHLI on viral release were next investigated by a viral release inhibition assay. Briefly, infected cells were washed and cultured in an infection medium for another 2 h, in the absence or presence of different concentrations of test compounds. The progeny viruses were then harvested and titrated. Both CHLA and CHLI inhibited virus release in a dose-dependent manner, like the positive control oseltamivir phosphate (Figure 4A).

It has been well documented that the influenza viral NA activity contributes to progeny virus release from an infected cell surface (Du et al., 2019). We therefore examined whether CHLA and CHLI blocked viral release of IAV by inhibiting viral NA activity. A standard fluorimetric method was used (Li et al., 2016), and the results showed that both CHLA and CHLI inhibited viral NA activity in a dose-dependent manner (Figure 4B).

Together, these results indicate that CHLA and CHLI inhibit IAV replication by preventing the viral NA-mediated progeny virus release.

### CHLA and CHLI Are Effective to Inhibit an Oseltamivir-Resistant IAV

**FIGURE 5:**
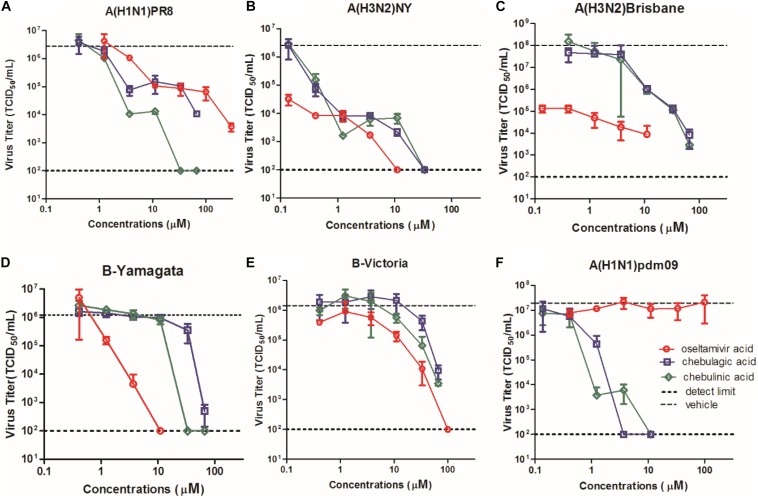
Dose–response curves of CHLA, CHLI, and reference compound oseltamivir acid against virus replication in a yield reduction assay. MDCK cells in 24-well plates were infected with **(A)** A/H1N1/PR8, **(B)** A/H3N2/NY, **(C)** oseltamivir-resistant influenza virus A/H1N1/pdm(09), **(D)** A/H3N2/Brisbane, **(E)** B-Yamagata, and **(F)** B-Victoria at an MOI of 0.01. The culture supernatants were collected at 24 h p.i., and virus titers (TCID_50_/mL) in the culture supernatants were determined using MDCK cells. Each point represents the mean and standard deviation of three independent experiments.

To compare the antiviral potency of CHLA and CHLI with the marketed NA inhibitor oseltamivir carboxylate, a yield reduction assay was performed with six influenza virus strains, including A/H1N1/PR8, A/H3N2/NY, A/H3N2/Brisbane, B-Yamagate, B-Victoria, and an oseltamivir-resistant A/H1N1pdm(09), which contains the NA/H274Y substitution. As a result, CHLA and CHLI showed significant inhibition against all influenza virus strains (Figure 5). Note that the IC_90_s of CHLA (1.26 μM) and CHLI (1.58 μM) upon A/H1N1/PR8 were lower than that of oseltamivir carboxylate (3.92 μM), suggesting their comparable activities (Figure 5A). While compared to IAVs (Figures 5A–C), IBVs showed less sensitivity to CHLA and CHLI (Figures 5D,E).Interestingly, NA/H274Y in A/H1N1/pdm(09) virus conferred resistance to oseltamivir carboxylate, but the potency of CHLA and CHLI was not affected by the substitution (Figure 5F).

## Discussion

In this study, a phenotypic screening approach was initially used to evaluate 352 natural product samples for activity against IAV infection, and 12 natural product samples were identified as putative hits with high anti-IAV potency. Among these hit natural product samples, unripe and ripe pods of *T. chebula* displayed the most potent anti-IAV activity.

Unripe and ripe pods of *T. chebula* are two different medical materials usually prescribed in Traditional Chinese Medicine formula. However, the compositions of unripe pods and ripe pods of *T. chebula* are highly overlapping, and our study showed that CHLA and CHLI, two overlapped tannin constituents of the two samples, exhibit highly inhibitory effects against IAV replication, with IC_50_s of 1.36 and 1.86 μM, respectively (Figure 2). Moreover, the SIs of CHLA and CHLI against IAV replication in MDCK cells are > 74 and > 54, respectively, suggesting that the two compounds are good candidates for novel antivirals against IAV (Figure 2).

Previous studies reported that CHLA and CHLI exhibit broad-spectrum antiviral activities by targeting viral glycoprotein–glycosaminoglycan interactions (Lin et al., 2011, 2013; Kesharwani et al., 2017). However, our data demonstrated that CHLA and CHLI do not interfere with influenza virus entry or RNA replication, but act as NA inhibitors targeting virus release.

Neuraminidase is a major surface glycoprotein with a sialidase activity, which contributes to the release of newly formed virions from infected cells and facilitates propagation of the virus (Du et al., 2019). For the purpose of antiviral development, NA is considered one of the major targets (Colman, 1994; Grienke et al., 2012). Currently, four NA inhibitors are available for the prophylaxis and treatment of influenza virus infections, including oseltamivir (Shobugawa et al., 2012), zanamivir (von Itzstein et al., 1993), peramivir (Alame et al., 2016), and laninamivir (Kashiwagi et al., 2016). Among these drugs, oseltamivir remains a first-line therapy since it was approved in 1999. However, resistance to oseltamivir has constantly been reported due to its wide use in clinic, and various mutants with resistance to the other NA inhibitors have also appeared (Samson et al., 2013; van der Vries et al., 2013; Lackenby et al., 2018). Therefore, it is imperative to discover novel NA inhibitors with different structures and mechanisms of action.

There are 11 NA subtypes of IAV, of which N10 and N11 were recently identified in bat IAV genomes (Tong et al., 2012, 2013). The N1–N9 subtypes can be phylogenetically divided into two groups. Group I includes N1, N4, N5, and N8, while group II is comprised of N2, N3, N6, N7, and N9 (Russell et al., 2006). Crystallographic studies show that group 1 NAs (except NA of A/H1N1pdm[09]) possess a large cavity termed 150-cavity adjacent to the catalytic site, but the 150-cavity was not observed in group 2 NAs (Li et al., 2010; Air, 2012). Upon binding of oseltamivir or zanamivir, the open 150-loop of group 1 NAs would adopt a closed conformation (Russell et al., 2006). Besides, inhibitors targeting the 150-cavity may also effectively target group 2 influenza NAs, perhaps by inducing the rigid closed 150-loop of group-2 NAs into a half-open one (Wu et al., 2013). The structure of A/H1N1pdm(09) NA presents a deficient 150-cavity in its crystal structure, conferring resistance to oseltamivir (Li et al., 2010). In addition, another auxiliary binding site adjacent to the sialic acid binding site called “430-cavity” has also been implicated and exploited for the design of new antivirals (Amaro et al., 2007; Swaminathan et al., 2013; Jia et al., 2019).

**FIGURE 6:**
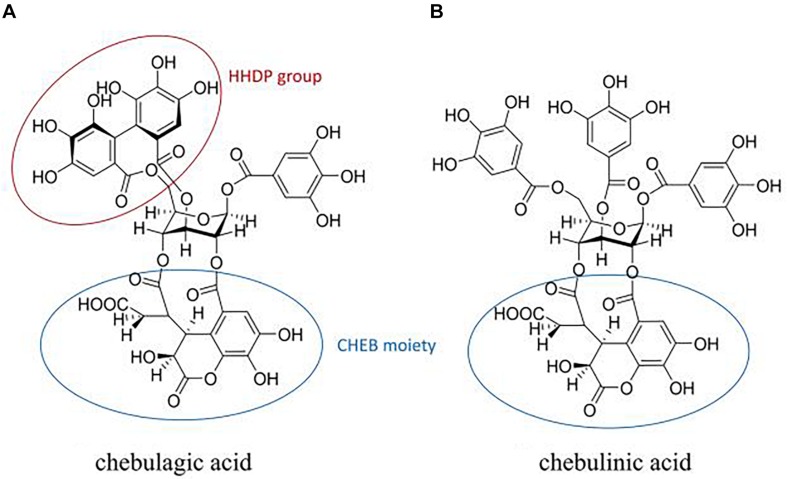
Structures of chebulagic acid **(A)** and chebulinic acid **(B)**. Circles are framed hexahydrodiphenoyl (HHDP) and chebuloyl (CHEB) moieties.

CHLA (1-*O*-galloyl-2,4-*O*-chebuloyl-3,6-*O*-hexahydroxydiph enoyl-β-D-glucose) (Figure 6A) and CHLI (1,3,6-tri-*O*-galloyl-2,4-*O*-chebuloyl-β-D-glucopyranoside) (Figure 6B) share the same molecular backbone of a β-D-glucose residue linked to a chebuloyl moiety. The difference is that CHLI has three free galloyl groups attached to the β-D-glucose residue, while CHLA contains only one galloyl group and a hexahydrodiphenoyl (HHDP) group, which is assumed to be a substitution for the two galloyl groups (Olennikov et al., 2015). The presence of the constrained HHDP group in CHLA is considered to result in larger spatial hindrance and less molecular flexibility. Thus, CHLI shows more broad-spectrum and potent biological activities due to the more favorable structure for entering the binding cavities or catalytic pockets of target proteases or enzymes. However, in our screening of anti-influenza activity described above, both of them showed excellent biological activity. The IC_90_ of CHLI is even better. It can be inferred that the active site is related to its common CHEB structure. Although the mechanism of action of CHLA and CHLI on NA is not clear yet, we predict that they bind to the active site(s) of NA, either one single interaction with the 430-cavity or multiple interactions to both 150- and 430-cavities.

## Conclusion

In summary, we demonstrate that CHLA and CHLI can effectively inhibit IAV replication. These compounds act as NA inhibitors and show antiviral potency to both wild-type and oseltamivir-resistant IAV strains. Therefore, they may be further developed as a potential therapy against IAVs.

## Data Availability Statement

The raw data supporting the conclusions of this article will be made available by the authors, without undue reservation, to any qualified researcher.

## Author Contributions

RD, LR, and QC conceived and designed the experiments. PL, YW, XH, XZ, LW, and RD performed the experiments. PL, PZ, and XL analyzed the data. RD, LR, and QC wrote the manuscript. All authors contributed to the final version.

## Conflict of Interest

The authors declare that the research was conducted in the absence of any commercial or financial relationships that could be construed as a potential conflict of interest.
