# Supplementary material for: Identification of Chebulinic Acid and Chebulagic Acid as Novel Influenza Viral Neuraminidase Inhibitors
Source: Front Microbiol. 2020 Feb 28;11:182. doi: 10.3389/fmicb.2020.00182 (PMC7093024; doi:10.3389/fmicb.2020.00182)
Supplement: Supplementary file 1 [file Data_Sheet_1.PDF]

Supplementary data

Table S1. Anti-IAV activities of 12 traditional Chinese medicinal materials.

Chinese Traditional Medical samples	IC_{50} (μg/mL)[a]	CC_{50} (μg/mL)[b]	SI[c]
Sargentodoxa cuneata (Oliv.) Rehd. et Wils.	21.2±1.4	>500	>23.6
Rosa rugosa Thunb.	17.6±0.9	343.3±129.9	19.5
Albizzia julibrissin Durazz.	21.6±4.9	211.3±9.7	9. 8
Dryopteris Crassirhizoma Nakai.	36.8±4.7	>500	>13.6
Paeonia lactiflora Pall.	34.8±2.4	>500	>14.4
Fagopyrum dibotrys (D. Don) Hara.	19.9±1.8	>500	>25.1
Acacia catechu (L.f.)Willd.	13.1±0.8	275.5±38.2	21.0
Unripe pods of *T. chebula*	5.8±1.4	255.1±1.3	44.0
Spatholobus suberectus Dunn	5.7±0.1	255.9±31.7	44.9
Nelumbo nucifera Gaertn.	11.6±8.6	>500	>43.1
Areca catechu L.	11.9±2.5	339.2±28.4	28.5
ripe pods of *T. chebula*	7.0±1.0	>500	>71.4

[a] IC_{50}, 50% inhibitory concentration;
[b] CC_{50}, 50% cytotoxic concentration;
[c] SI, Selectivity index.

S1 High-Performance Liquid Chromatography (HPLC) Analysis of CHLA and CHLI in unripe and ripe pods of *T. Chebula*

Dispensing Granules of both unripe and ripe pods of *T. Chebula* (equivalent to 0.1 g raw material）were dissolved in 100 mL methanol. The solution was filtered through 0.45 μm membranes before HPLC analysis. The contents of CHLA and CHLI in unripe and ripe pods of T. Chebula were determined by Agilent series 1260 HPLC-DAD instrument (Agilent Technologies, Santa Clara, CA, USA). Chromatographic separation was carried out at 25 oC on a ZORBAX SB-C18 column (5 μm, 4.6 × 250 mm, Agilent Technologies). The mobile phase was composed of A (Methanol) and B (0.1% Phosphate water), a gradient elution was performed as follows: 0-8 min, 10-20% A; 8-15 min, 20-35% A; 15-40 min, 35-50% A; 40-50 min, 50% A. The sample injection volume was 10 μL, and the flow rate was 1.0 mL/min. The DAD detector scanned from 200 to 500 nm, and the samples were detected at 254 nm.

As Figure S1shows, CHLA and CHLI are constituents of both unripe and ripe pods of *T. Chebula*: the unripe pods of *T. Chebula* contains 9.380mg/g of CHLA and 9.970mg/g of CHLI, while the ripe pods of *T. Chebula* contains 14.155 mg/g of CHLA and 11.785 mg/g of CHLI.

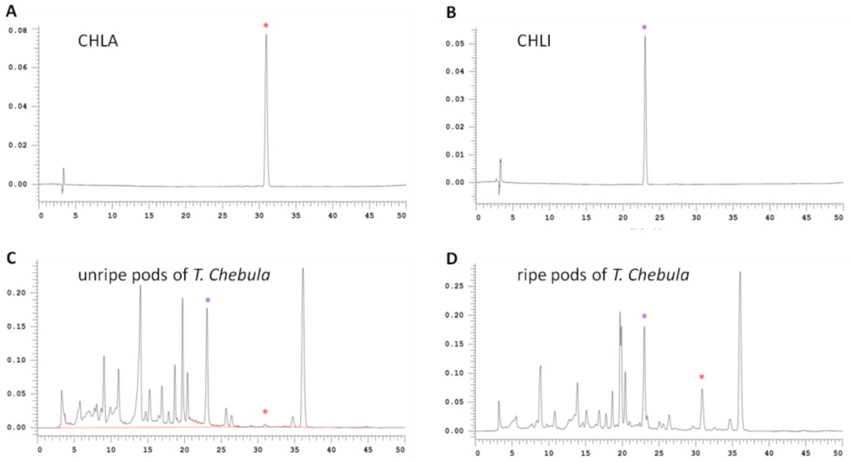

Figure S1. HPLC analysis of (A) CHLA, (B) CHLI, (C) unripe pods of T. Chebula and (D) ripe pods of *T. Chebula*. Red * indicates the peak of CHLA, while purple * indicates the peak of CHLI.

S2. Cell cytotoxicity for both CHLA and CHLI in one-cycle infection inhibition assay

MDCK cells growing in 96-well plate were infected with PR8-PB2-Gluc at an MOI of 0.1 in presence of various concentrations of test samples/compounds. After 24 hrs incubation, cell cytotoxicity was examined.

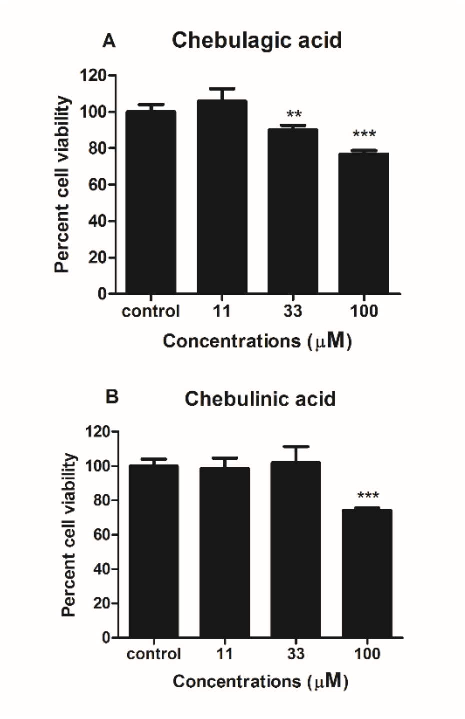

Figure S2. Cell cytotoxicity for both CHLA and CHLI in one-cycle infection inhibition assay. Data are means ± SD from three independent experiments. **, p<0.01, ***, p<0.001; student's t test.
